# Cancer Patients' Views on Ultrahypofractionated Radiotherapy: A Questionnaire-Based Survey

**DOI:** 10.7759/cureus.72093

**Published:** 2024-10-22

**Authors:** Koyo Kikuchi, Takafumi Segawa, Hirobumi Oikawa, Yoshiro Ieko, Ryuji Nakamura, Hisanori Ariga

**Affiliations:** 1 Department of Radiation Oncology, Iwate Medical University, Iwate, JPN; 2 Department of Radiation Oncology, Iwate Prefectural Central Hospital, Iwate, JPN; 3 Department of Radiology, Morioka Red Cross Hospital, Iwate, JPN

**Keywords:** alternative schedule, questionnaire survey, stereotactic body radiotherapy, ultrahypofractionated radiotherapy, weekly radiotherapy

## Abstract

Introduction: Ultrahypofractionated (UHF) radiotherapy provides several treatment schedules, including five fractions per week (5/5-UHF), three fractions per week (3/5-UHF), and one fraction per week (1/5-UHF). This study aimed to assess patient preferences for these UHF radiotherapy schedules and offer insights to support patient-centered radiotherapy.

Methods: A questionnaire survey was conducted among cancer patients who had received at least 10 fractions of definitive or palliative radiotherapy, delivered on consecutive weekdays at our institution. The survey was administered during the final week of treatment and included four questions regarding the patients' living conditions and seven questions about their perceptions of radiotherapy, including preferences for UHF radiotherapy schedules.

Results: Between April and July 2023, 71 eligible patients completed the questionnaire. The mean age was 65.2 years; 48 patients (68%) were male, and 36 patients (51%) were inpatients. Thirty-one patients (44%) traveled more than one hour to the hospital, and 34 patients (47%) reported stress from daily hospital visits for radiotherapy. In response to the question, "If you were to receive 5-fraction radiotherapy, would you choose 5/5-UHF, 3/5-UHF, or 1/5-UHF?" 64 patients responded. Among these, 27 (42%) chose 5/5-UHF, 19 (30%) chose 3/5-UHF, and 18 (28%) chose 1/5-UHF. Regarding the question, "Which is more important: a shorter treatment period with the same fractionation or reduced fractionation with the same treatment period?" 31 patients (48%) prioritized shorter treatment periods, while 12 (19%) preferred reduced fractionation. Although approximately half of the patients expressed distress from consecutive days of radiotherapy, the shortest treatment period was generally preferred, even if it involved consecutive sessions. The 1/5-UHF radiotherapy schedule was particularly popular among younger male patients, those with longer travel times, and those working full-time.

Conclusion: These findings suggest that tailoring radiotherapy schedules to patients' social circumstances may enhance overall satisfaction with treatment.

## Introduction

The standard schedule for radiotherapy is daily treatment from Monday to Friday, excluding weekends and holidays (conventional fractionated (CF) radiotherapy: 1.8-2.0 Gy/fraction, one fraction/day, five days/week). Since the coronavirus disease 2019 (COVID-19) pandemic, moderate hypofractionated (MHF) radiotherapy (2.5-3.4 Gy/fraction [1]) and ultrahypofractionated (UHF) radiotherapy (> 5 Gy/fraction [1]), which can reduce the frequency of visits and contact opportunities, have increased in prevalence. For UHF radiotherapy, daily treatment (5/5-UHF) is not always used as in CF radiotherapy, but rather every other day (3/5-UHF) or weekly (1/5-UHF), based on practice and biological evidence.

For example, the FAST trial [2] demonstrated the non-inferiority of 28.5 Gy in five once-weekly fractions (1/5-UHF) over five weeks compared to CF radiotherapy in postoperative breast cancer irradiation. Similarly, the FAST-Forward trial [3] showed the non-inferiority of 26 Gy in five fractions over one week (5/5-UHF) compared to MHF radiotherapy in the same setting. In curative radiotherapy for prostate cancer, 5/5-UHF, 3/5-UHF, and 1/5-UHF schedules exist [1,4-6], but five-fraction prostate UHF radiotherapy with consecutive daily treatments is not recommended because of a potential increased risk of late urinary and rectal toxicity [1]. In the randomized phase II trial comparing 3/5-UHF with 1/5-UHF for prostate UHF radiotherapy, there was no difference in toxicity between the two schedules [6]. In the treatment of early-stage lung cancer, UHF radiotherapy is delivered either as 5/5-UHF or 3/5-UHF, but regardless of which schedule is used, the local control rate and overall survival rate are the same [7]. In the treatment of hepatocellular carcinoma with UHF radiotherapy, 3/5-UHF has been reported to be related to less treatment-related fatigue than 5/5-UHF [8].

A 1/5-UHF schedule is available for patients, such as the elderly or frail, who face challenges in attending daily hospital visits. In postoperative radiotherapy for uterine cancer, a regimen of 30 Gy delivered in five fractions over five weeks has been documented using the 1/5-UHF radiotherapy schedule [8]. Similarly, for elderly bladder cancer patients unable to undergo daily treatments, a 1/5-UHF protocol of 36 Gy in six fractions over six weeks has been reported [9]. Furthermore, in palliative radiotherapy for head and neck cancer, a 1/5-UHF regimen of 24 Gy delivered in three fractions over four weeks has also been reported [10].

Thus, a variety of schedules ranging from one to five times per week are reported for UHF, but the differences in efficacy and toxicity between the schedules are still unclear. Therefore, patients should have some opportunity to choose their schedule. Patients undergoing radiotherapy were surveyed to determine their scheduling preferences for radiotherapy. In particular, the questions focused on whether 1/5-UHF was preferred by the patients.

## Materials and methods

This questionnaire-based survey of patients who received external beam radiotherapy at our hospital was approved by the Institutional Review Board of Iwate Medical University, Iwate, Japan (approval number: MH2022-175). The hospital is an academic medical center located on the outskirts of a city with a population of 300,000 and limited public transportation. The questionnaire comprised four questions (Q1-4) regarding the burden of hospital visits and six questions (Q5-10) regarding patients’ perceptions of external beam radiotherapy. For Q7-8 and Q10, patients were asked to choose an answer on a scale of 0 to 10. The inclusion criteria were as follows: (i) received external beam radiotherapy for malignant tumors at our institution for at least 10 consecutive weekdays; (ii) provided written informed consent; and (iii) completed the questionnaire by themselves. Patients were excluded from the study if the physician determined that they were unsuitable for the questionnaire survey. The questionnaire was administered after the patients had received at least five fractions and the physician explained the purpose and methodology of the study. Questionnaires were returned by patients during the last week of radiotherapy. At our hospital, we provide radiotherapy to approximately 800 patients annually. A survey was conducted with a target sample size of 86 derived from this population, using a margin of error of 10%, a confidence level of 95%, and an assumed standard deviation of 50%.

## Results

Between April and July 2023, responses from 72 patients were collected. One of these patients did not meet the eligibility criteria because their treatment was not conducted daily and was therefore excluded from the analysis. The remaining 71 patients met the eligibility criteria and were included in the analysis. All 71 patients completed the scheduled radiotherapy without any interruptions.

Patients

Patient backgrounds are shown in Table 1. When compared with data from the Japanese Radiation Oncology Database (JROD) 2022 [11], our patients showed deviations in the proportion of men (68% vs. 53%), hospital treatment (51% vs. 34%), palliative treatment (11% vs. 36%), and irradiated sites of the head and neck (28% vs. 14%).

**Table 1:** Patient background ECOG-PS: Eastern Cooperative Oncology Group Performance Status

Parameters	n=71
Mean age (years)	65.2
Gender, male	48 (68%)
Primarily treated in an inpatient setting	36 (51%)
Stage	
I–II	32 (45%)
III–IV	37 (52%)
Brain tumor	2 (3%)
ECOG-PS	
0–1	67 (94%)
2–4	4 (6%)
Radiotherapy intent	
Radical/perioperative	63 (89%)
Palliative	8 (11%)
Fractionation	
10–20	24 (34%)
21–30	45 (63%)
>30	2 (3%)
Treatment site	
Brain	2 (3%)
Head and neck	20 (28%)
Chest	13 (18%)
Abdomen	11 (16%)
Pelvis	25 (35%)

Questionnaire and answers expressed in distribution

To the first question, "How long does it take to reach the hospital from home?", 15 (21%) answered <0.5 hours, 25 (35%) said 0.5-1.0 hours, 22 (31%) said 1.0-2.0 hours, and nine (13%) said >2 hours.

**Figure 1:**
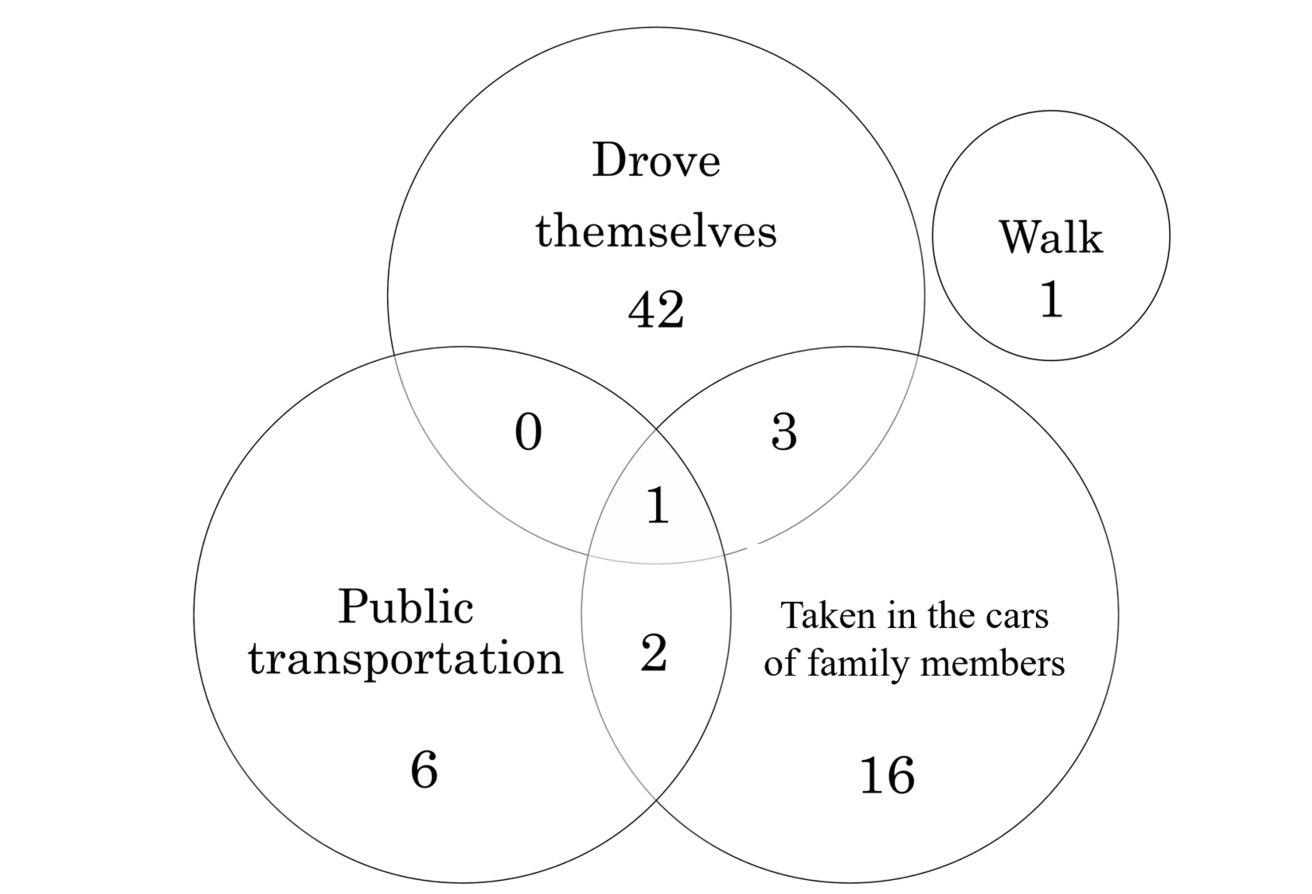
A diagram showing the number of patients divided by the modes of transports for visiting the hospital

The second question was "How do you travel to the hospital?". Forty-six drove themselves, 22 were taken in the cars of family members, nine traveled by public transportation, and one reached on foot. Six patients selected multiple choices. The distribution is presented in Figure 1.

The third question was "How many people do you live with?". Six (9%) patients lived alone; 34 (48%), with one family member; 18 (25%), with two; eight (11%), with three; and five (7%), with more than four people.

The fourth question was "What is your employment status?". Thirty-one (44%) patients were unemployed; 16 (23%) were full-time employees; four (6%), were part-time employees; five (7%), were contract employees; and six (8%), were domestic workers. The remainder (nine (13%)) selected “other” with remarks indicating self-employed, retired employee, full-time childcare, or student.

**Figure 2:**
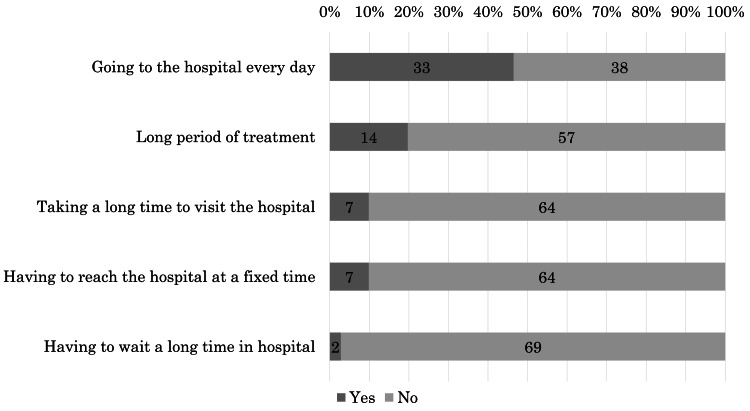
Factors leading to distress during the radiotherapy course

The fifth question was, "What made you distressed during the radiotherapy course? Please select all that apply." Forty-nine (69%) patients reported experiencing stress from the following factors: 33 (46%), going to the hospital every day; 14 (20%), long treatment period; seven (10%), taking a long time to visit the hospital; seven (10%), having to reach the hospital at a fixed time; and two (3%), having to wait a long time for radiotherapy (Figure 2). The remaining 22 (31%) patients expressed the absence of stress, nine left all choices unselected, and 13 selected "other" with remarks unrelated to the treatment schedule, such as rectal and bladder preparation for prostate radiotherapy.

**Figure 3:**
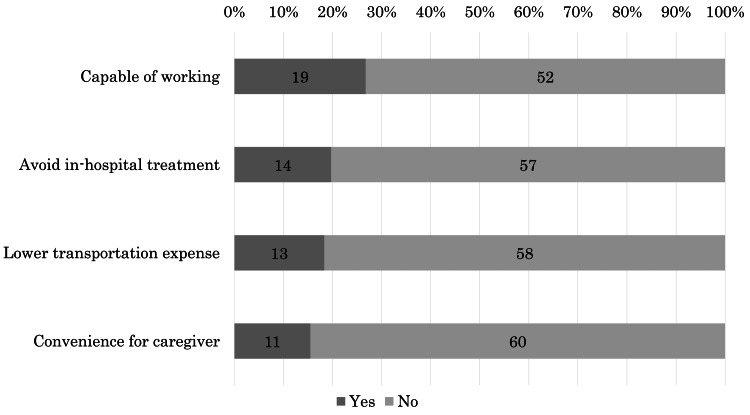
Advantages for patients if the radiotherapy schedule was 1/5-UHF instead of CF CF: conventional fractionated radiotherapy; 1/5-UHF: ultrahypofractionated radiotherapy one fraction per week

The sixth question was, "What would have made you comfortable if CF radiotherapy had been converted to 1/5-UHF? Please select all that apply." Fifty-seven (80%) found some advantages of 1/5-UHF; 19 (27%) would be capable of working; 14 (20%) wanted to avoid in-hospital treatment; 13 (18%) thought they would have had lower transportation expenses; and 11 (15%) expressed that it would be convenient for caregivers (Figure 3). Other comments included that it reduced their physical stress, made it easier to run errands, and allowed them to spend more time at home. The remaining 14 (20%) patients found no advantage in 1/5-UHF: 11 selected "good for nothing," one selected "other" with a remark of "not good,” whereas two did not select anything.

**Figure 4:**
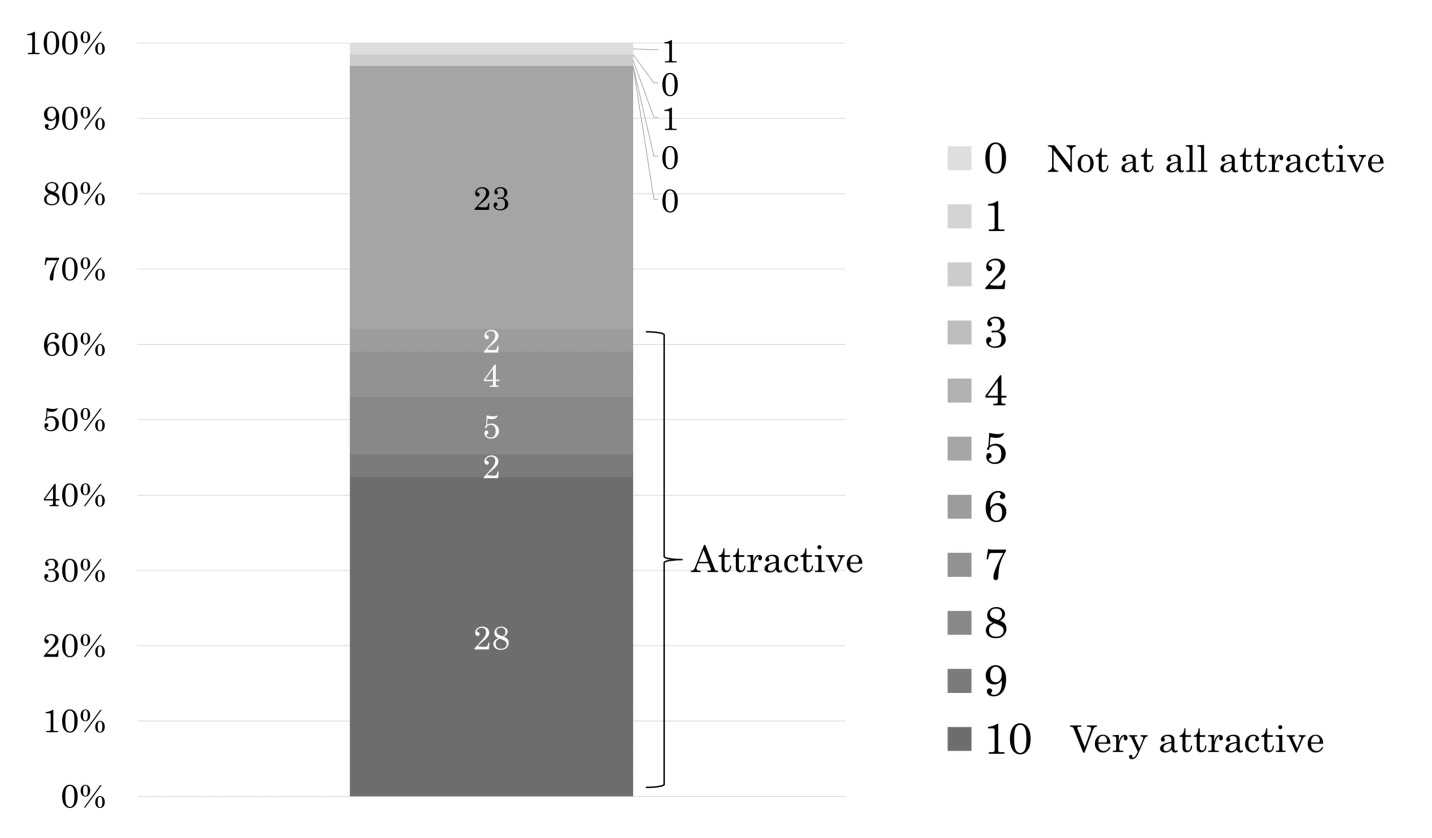
Proportion of patients who found it attractive if the schedule was 1/5-UHF instead of CF with the same treatment period With 0 representing "not at all attractive" and 10 representing "very attractive," patients rated the degree of attractiveness on an 11-point scale from 0 to 10. CF: conventional fractionated radiotherapy; 1/5-UHF: ultrahypofractionated radiotherapy one fraction per week

The seventh question was, "If you received radiotherapy by 1/5-UHF instead of CF radiotherapy for the same period of treatment, how attractive would 1/5-UHF be to you?" ("If you were to receive three weeks of radiation with five fractions per week, would you find three weeks of radiation with one fraction per week attractive?" It is assumed that the time per treatment, treatment efficacy, and side effects are equivalent for both schedules). Sixty-six of the 71 patients answered this question. Forty-one (62%) responded “attractive,” with 28 of them rating it as "very attractive.” Two patients (3%) responded as “unattractive” or "not at all attractive,” and 23 patients (35%) expressed neutrality (Figure 4).

The eighth question was, "If the radiotherapy you received was 1/5-UHF instead of CF, and the period of treatment was five to six weeks (five to six fractions), would you find 1/5-UHF attractive?" (It is assumed that the time per treatment, treatment efficacy, and side effects are equivalent for both schedules).

**Figure 5:**
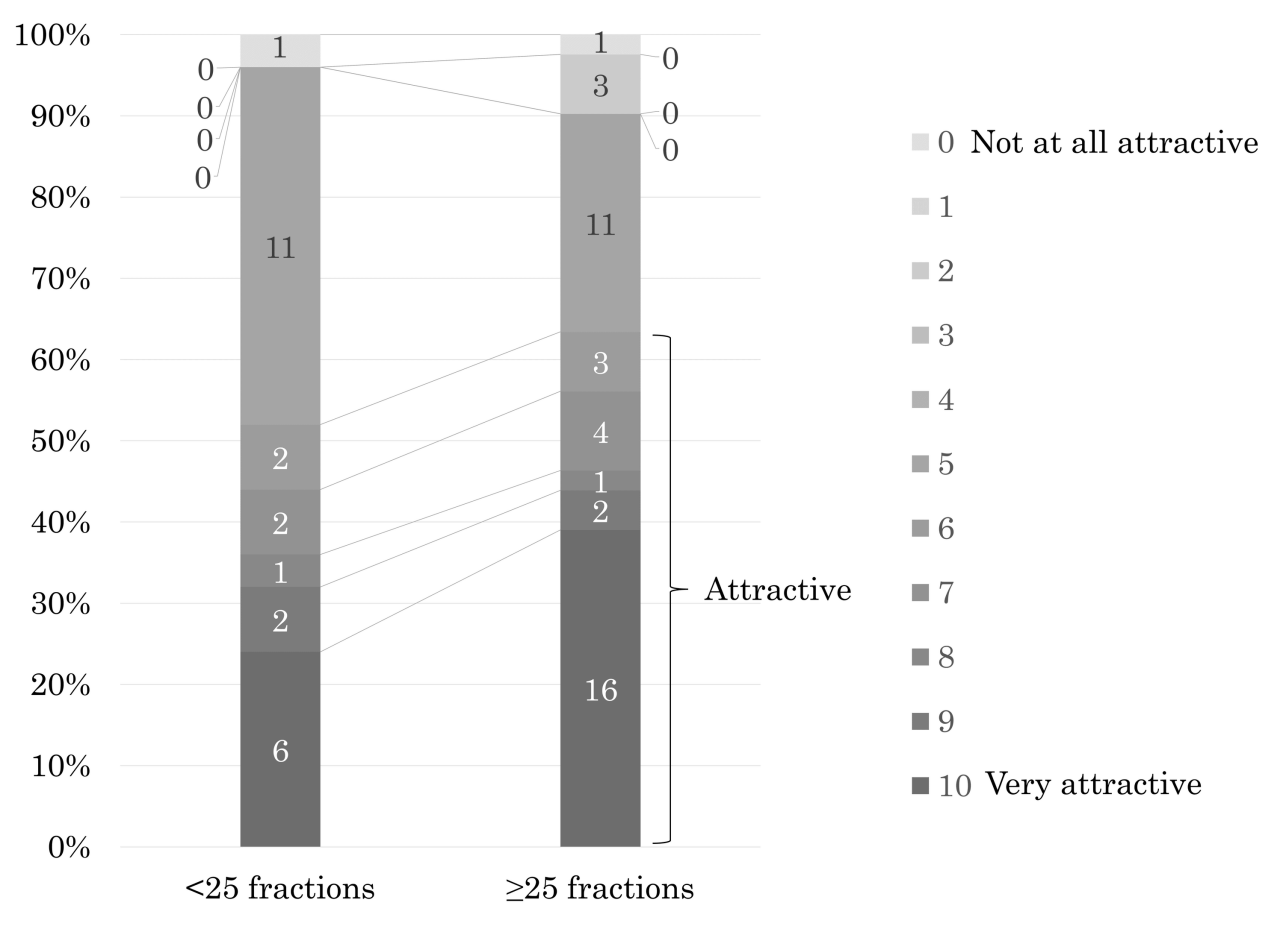
The proportion of patients who found it attractive if the schedule was 1/5-UHF instead of CF with the 1/5-UHF schedule over a period of five to six weeks was compared between those who received <25 and ≥25 fractions. With 0 representing "not at all attractive" and 10 representing "very attractive", patients rated the degree of attractiveness on an 11-point scale from 0 to 10. CF: conventional fractionated radiotherapy; 1/5-UHF: ultrahypofractionated radiotherapy one fraction per week

Twenty-five of the 27 patients who received radiotherapy with fewer than 25 fractions answered this question. Thirteen of the 25 (52%) patients responded “attractive,” of which six rated it as “very attractive.” One of the 25 (4%) patients responded, “not attractive at all.” Eleven of the 25 (44%) patients expressed neutrality. Forty-one of the 44 patients who received radiotherapy with more than 25 fractions answered this question. Twenty-six of the 41 (63%) patients responded “attractive,” with 16 of them rating it as “very attractive.” Four of the 41 (10%) patients responded “unattractive,” of which one rated it as "not attractive at all." Eleven of the 41 (27%) patients expressed neutrality (Figure 5).

**Figure 6:**
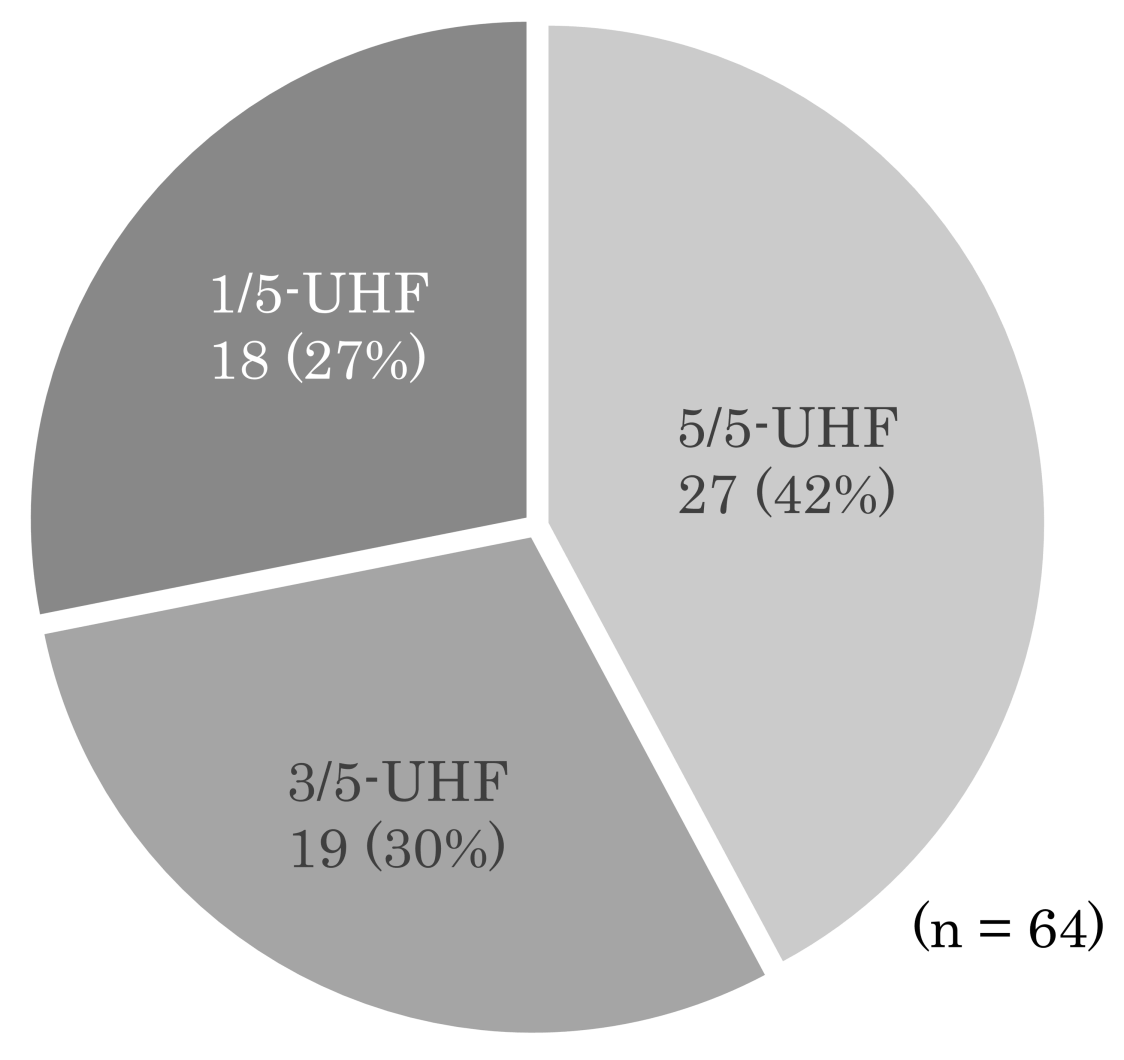
Number of patients who preferred 5/5-UHF for a week, 3/5-UHF for two weeks, or 1/5-UHF for five weeks if they were treated by UHF radiotherapy. n/5-UHF: ultrahypofractionated radiotherapy n fractions per week

The ninth question was, "If you were to receive five-fraction radiotherapy, would you choose 5/5-UHF, 3/5-UHF, or 1/5-UHF?". Sixty-four of the 71 patients answered this question, of whom 27 (42%) chose 5/5-UHF; 19 (30%) chose 3/5-UHF; and 18 (28%) preferred 1/5-UHF (Figure 6).

The tenth question was, "Which is more important for you, shorter treatment periods with the same fractionation or reduced fractionation with the same treatment periods?"

**Figure 7:**
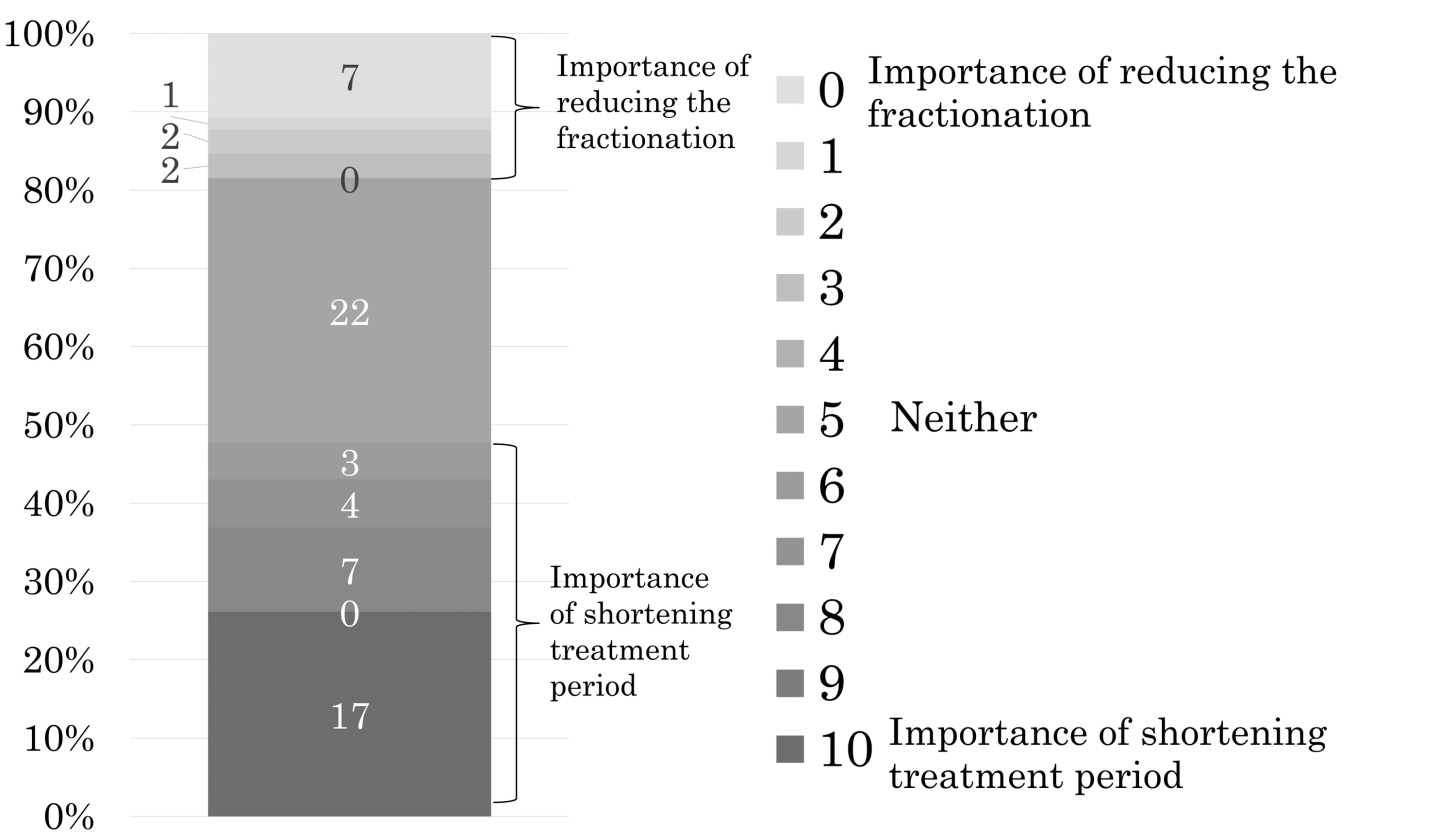
Weight in preference of patients for either shortening the treatment periods of radiotherapy or reducing the times of fractionation. With 0 representing "the importance of reducing the fractionation", five representing "neither", and 10 representing "the importance of shortening the treatment period", patients rated which they prioritized on an 11-point scale from 0 to 10.

Sixty-four of the 71 patients answered this question. Thirty-one of the 64 (48%) patients responded that shortening the treatment period was more important, with 17 indicating that it was the most important. Twelve of the 64 (19%) patients preferred reducing the number of fractions, with seven indicating that it was the most important. Twenty-two of the 64 (34%) patients responded "neither" (Figure 7).

Analysis of the backgrounds of patients who had a preference for 1/5-UHF

Patients who indicated a preference for 1/5 UHF in question number 9 were more likely to be younger, male, had a hospital visit time >1 hour, had a full-time job, and were outpatients (Table 2).

**Table 2:** Comparison of backgrounds of patients who prefer 1/5-UHF and 5/5-UHF or 3/5-UHF ECOG-PS: Eastern Cooperative Oncology Group Performance Status; n/5-UHF: ultrahypofractionated radiotherapy n fractions per week

Parameters	5/5-UHF (n=27)	3/5-UHF (n=19)	1/5-UHF (n=18)
Median age (range)	67 (16-82)	70 (39-80)	63.5 (40-82)
Male	16 (59%)	13 (68%)	14 (78%)
Hospital visit time > 1 hour	12 (44%)	6 (32%)	10 (56%）
Median number of cohabitants (range)	2 (1-5)	3 (1-5)	2 (1-5)
Full-time employee	7 (26%)	3 (16%)	6 (33%)
Outpatient hospitalization	11 (41%)	8 (42%)	11 (61%)
ECOG-PS ≥1	8 (30%)	4 (21%)	5 (28%)
Stage ≥ III	11 (41%)	12 (63%)	11 (61%)
Median fractionation (range)	30 (10-33)	28 (10-33)	25 (12-39)

## Discussion

Radiotherapy offers the advantage of being more convenient for patients compared to other treatment modalities as it can often be administered on an outpatient basis. However, travel time to cancer centers can vary significantly depending on factors such as transportation, socioeconomic status, and regional characteristics [12]. For patients treated at suburban hospitals with inadequate public transportation, like our hospital, the greatest burden often lies in the daily commute. In this study, half of the patients traveled more than one hour by car, either driving themselves or being driven by a family member. This created a significant burden in terms of time, transportation costs, and the physical and emotional toll on the family member providing the transport. Additionally, 57% of the patients lived alone or with only one family member, making it difficult to receive support from their families. While hospitalization was an option for those facing such challenges, it required taking time off work. In this study, 23% of the patients were employed full-time, which highlights the need to consider the social and work-related factors affecting these patients. In this regard, UHF radiotherapy offers a clear advantage over CF radiotherapy, as it can reduce the burden of commuting to and from the hospital. This is particularly important in Japan, where an increasing number of people live alone due to a declining birthrate and aging population, and where the absence of family support may lead to delays or withholding of treatment [13].

Ultrahypofractionated radiotherapy can be administered on various schedules, ranging from once a week to five times a week. Although no clear consensus exists on the differences in efficacy and safety between these schedules, studies have shown no significant difference in overall efficacy [6-8]. However, there is a tendency for fewer side effects to be reported when treatments are given every other day rather than daily [1,8]. This study assumed no differences in efficacy or side effects between UHF radiotherapy schedules and aimed to determine whether patients preferred non-daily radiotherapy, even if it resulted in longer overall treatment duration, or whether they favored daily treatments with the shortest total treatment time. Specifically, the study examined the potential demand for 1/5-UHF, which has an overall treatment duration similar to CF radiotherapy.

In this study, 69% of patients who had undergone CF radiotherapy reported feeling stressed by the daily radiotherapy schedule. Many of these patients indicated that weekly treatment would be appealing, even if the overall treatment period remained the same as CF radiotherapy. On the other hand, patients seemed to prioritize a shorter overall treatment time over the number of fractionations, with many expressing a desire to minimize the total duration of treatment, even if they were receiving daily radiotherapy. Conversely, 58% of patients favored non-consecutive fractionations such as 1/5-UHF and 3/5-UHF. While not definitive, there was a tendency for weekly treatments, such as 1/5-UHF, to be preferred by men who worked full-time and had long commutes to the hospital. Full-time workers may prefer treatment on certain days of the week when their workload is lighter, rather than taking an entire week off. These varied patient preferences suggest the importance of offering alternatives in UHF radiotherapy to enhance patient satisfaction, particularly in the era of "patient-centered care" in radiation oncology [14]. The healthcare delivery system must evolve to meet these demands, incorporating a shift from "cure-seeking care" to "cure- and support-seeking care" [15,16].

Although UHF radiotherapy is an attractive option for patients, its indications remain limited, and CF radiotherapy remains the standard radiotherapy approach for many diseases. When patients are unsuitable for daily or long-term treatment, UHF radiotherapy may be suggested. However, after thorough discussions regarding the uncertainties and potential for acute toxicity, many patients opted against UHF radiotherapy due to concerns about increased toxicity and uncertain long-term outcomes [17]. It is also important to note that during the COVID-19 pandemic, numerous articles advocated for hypofractionated schedules for major disease sites, but the quality of evidence in these articles was often lower than that supporting established dose fractionation schedules [18].

This study has several limitations. First, it included patients receiving radiotherapy at an academic medical center specializing in advanced cancer treatment, where many patients received multimodal treatments requiring hospitalization. In particular, patients with head and neck cancers, which are relatively common at our hospital, were hospitalized for chemoradiotherapy. Therefore, it is important to recognize that the diseases and treatments of these patients may differ from those in general hospitals. Additionally, few patients in this study received palliative radiotherapy, which might influence treatment goals and preferences, with patients focusing on fewer weekly doses and shorter treatment durations. This may have led to an overestimation of the preference for UHF radiotherapy. Furthermore, the survey sample was not large enough to provide definitive conclusions regarding which patient backgrounds were associated with different schedule preferences. Lastly, the survey included patients for whom hypofractionated radiotherapy was not a realistic option.

This study highlights patient preferences regarding radiotherapy schedules. Offering comprehensive information about scheduling options when performing UHF radiotherapy can facilitate better decision-making by patients and improve physician-patient communication, ultimately enhancing patient satisfaction with their treatment.

## Conclusions

Patients have some problems with their radiotherapy schedules, and we need to resolve these issues. Ultrahypofractionated radiotherapy presents a promising solution by offering alternative schedules beyond standard daily treatments. This study examined patient preferences for UHF radiotherapy schedules and found that some patients prefer options such as every other day or weekly treatment instead of daily sessions. In the era of UHF radiotherapy, it is essential to develop schedules that effectively balance tumor control, safety, and patient convenience. Tailoring radiotherapy schedules to better align with patients' social circumstances can significantly enhance overall treatment satisfaction.
